# Mind the Gap: Bridging the Divide from Sequencing Data to Empiric Phenotypes in the Human Gut Microbiota

**DOI:** 10.1128/msystems.00207-22

**Published:** 2022-06-13

**Authors:** Ryan T. Fansler, Wenhan Zhu

**Affiliations:** a Department of Pathology, Microbiology, and Immunology, Vanderbilt University, Nashville, Tennessee, USA

**Keywords:** *Bacteroidetes*, human gut microbiota, glycan utilization, phenotypic assay, polysaccharide utilization

## Abstract

The gut microbiome exerts a powerful influence on human health and disease. Elucidating the underlying mechanisms of the microbiota’s influence is hindered by the immense complexity of the gut microbial community and the glycans they forage. Despite a wealth of genomic and metagenomic sequencing information, there remains a lack of informative phenotypic measurements. Pudlo NA, Urs K, Crawford R, Pirani A, et al. (mSystems 7: e00947-21, 2022, https://doi.org/10.1128/msystems.00947-21) decode this complexity by introducing a scalable assay to measure specific carbohydrate utilization in the dominant microbiota phylum *Bacteroidetes*. The results reveal a mosaic structure of glycan utilization, both genetic and functional, underpinning niche construction in the human gastrointestinal tract. This Commentary highlights the significance of their findings in connection to the field’s growing appreciation for competition, cooperation, and horizontal gene transfer in shaping the highly complex microbial community.

## COMMENTARY

The human gastrointestinal tract is home to a vastly complex microbial community termed the gut microbiota—an assembly of protozoa, archaea, fungi, viruses, and, by mass, predominantly bacteria (1). The microbiota is now appreciated to play pivotal roles in human health and disease, including influencing host metabolism (2), shaping immune system development (3), governing susceptibility to infections, and affecting a broad range of noncommunicable diseases (4, 5), such as inflammatory bowel disease (6). The functional capacity (what they can do) of the microbiota is influenced strongly by its composition (who is there), which, in turn, is determined primarily by the availability of nutrients and the capacity of the microbial community to consume them (2). The need to rationally modulate microbiota functions via dietary intervention has sparked intense interest in understanding how the nutrient landscape shapes the composition, and thus the functions, of the gut microbiome (7–10). While the guiding principle that intestinal bacteria feast on dietary and host-derived glycans in the distal intestine is well illustrated (11, 12), defining how specific glycans influence microbiome structure has remained a significant challenge in the field. Endeavors to illuminate which microorganisms consume which specific nutrients have been met with challenges in (i) the complexity of the gut bacterial community; (ii) culturing specific microbiota species; (iii) the immense chemical diversity of dietary, microbial, and host-derived glycans; and (iv) the impressive armament of glycan-degrading enzymes encoded by individual bacterial genomes (13). Pudlo et al. (14) offer the field a significant step in closing this gap. They present a scalable, high-throughput assay for measuring the carbohydrate utilization in over 300 members of *Bacteroidetes*, a dominant saccharolytic phylum of the human gut (15). This platform revealed characteristic roles among species, as well as a high level of diversity in carbohydrate utilization, comprising a mosaic genetic structure. The level of mosaic heterogeneity suggests the occurrence of interspecies horizontal gene transfer (HGT). Ten years in the making, this data set and the authors’ interpretations present a significant advance for the field of intestinal microbial interactomics.

To shed light on how specific nutrients sustain specific lineages in *Bacteroidetes*, Pudlo et al. developed a custom phenotyping array to characterize the carbohydrate utilization of 354 strains across 29 species in this prominent phylum. This data set alone represents a valuable resource for the gut microbiome field. Glycans are the most chemically diverse class of molecules in living systems. Glycans’ chemical diversity and abundance in the human diet make them a powerful driving force for the genome evolution and diversification of the microbiota. The authors elucidate the population distribution of functional polysaccharide utilization loci (PULs; or glycan utilization loci [GULs]) in the microbiota, providing phenotypic evidence to finely define mechanisms underlying niche specification within the gut. In doing so, they advance our understanding of the evolutionary past, present, and future of carbohydrate niches in the intestinal environment. The authors observed similar preferences for carbohydrate sources among closely related species, along with paradoxically high diversity in PUL distribution. Their findings complement the discovery by Park et al. that *Bacteroides* fitness is, in part, determined by context-dependent inhibition by the metabolite butyrate, depending on the specific sugars utilized by individual strains (16). Integrating these groups’ data sets could even further inform our understanding of carbohydrate utilization and its evolution in the intestine. Finally, while the purchase or custom purification of all of the required substrates could be significant barriers, the formulation/design of this growth assay is openly accessible and is applicable to additional members of the human gut microbiota, as well as to additional carbohydrates or other nutrients of interest. This assay, substantiated by its resultant data set, provides a much-needed roadmap in translating the highly sequenced-based data of the microbiome field into phenotype-based functional metabolomics, forming the basis of precision dietary modulation of the gut microbiome.

In analyzing *Bacteroidetes* PULs via pangenomic reconstruction, the authors discovered a highly diverse genetic landscape. The PUL-based clustering of species does not correlate to the overall *Bacteroidetes* phylogeny. Frye et al. observed a similar phenomenon among the vitamin B_12_ transport loci of *Bacteroidetes* (17). Both groups suggest the occurrence of interspecies HGT as a likely explanation. Indeed, Frye et al. demonstrated the existence of functional corrinoid transport loci on conjugative transposons within the phylum. Additionally, Hehemann et al. discovered integrative and conjugative elements (ICEs) carrying PULs that enable the catabolism of algal glycans, common in seaweed products, that likely originated from marine bacteria (18). However, Pudlo et al. did not observe any typical indicators of ICEs. Instead, they present evidence in favor of HGT based on intergenomic homologous recombination. This model would allow bacteria to share advantageous genes with closely related species while excluding more distant relatives without the requisite level of sequence homology required for recombination, “underscoring the notion that individual gut symbiont genomes are not just highly variable, but dynamically so.” Furthermore, the mosaicism revealed by this data set may inform our ecological understanding of the gut microbiome. As specific glycan utilization shapes the competition and cooperation among *Bacteroidetes*, this dynamic genome variability underpinning carbohydrate utilization could serve an integral role in the assembly and stability of the gut as an ecological system (19). Thus, while HGT has already been shown to be highly prevalent in the gut ecosystem (20), Pudlo et al., Frye et al., and Hehemann et al. together construct a model in which HGT plays an essential role in shaping nutrient competition, niche formation, and potential cooperation among microbiota populations. Ultimately, future studies of intestinal HGT may illuminate key processes that impact the composition and functions of the intestinal microbiota and, by extension, human health.

Pudlo et al. provide the field with an innovative growth assay and a resultant data set that contribute to the growing appreciation for the important role of HGT in the microbiome. Additional applications of this assay to new phyla or new nutrients promise to yield further understanding of this incredibly important microbial ecosystem. With a renaissance in culturing techniques and a shrinking gap between sequence data and phenotypic analyses, it is an exciting time to be involved in microbiome research.
